# Assessing Participation Among Stroke Survivors: A Systematic Review of Patient-Reported Outcome Measures

**DOI:** 10.1155/oti/4928745

**Published:** 2025-07-17

**Authors:** Benyamin Hamid, Mahnaz Hejazi Shirmard, Seyedeh Maryam Shafighi Kuzani, Marzieh Pashmdarfard

**Affiliations:** ^1^Student Research Committee, Department of Occupational Therapy, School of Rehabilitation, Shahid Beheshti University of Medical Sciences, Tehran, Iran; ^2^Department of Occupational Therapy, School of Rehabilitation, Shahid Beheshti University of Medical Sciences, Tehran, Iran

**Keywords:** cerebrovascular accidents, COSMIN, ICF, ischemia, measure, participation, PROM

## Abstract

**Introduction:** Stroke significantly impacts survivors' lives, affecting their physical, cognitive, and emotional well-being. Understanding how these individuals participate in daily activities and societal roles is crucial for optimal rehabilitation and support. Patient-reported outcome measures (PROMs) offer valuable insights into stroke survivors' experiences and perceptions regarding their participation in various life aspects. This systematic review focuses on PROMs specifically designed to evaluate participation following stroke, based on the International Classification of Functioning, Disability and Health (ICF). By analyzing existing measures, this review is aimed at identifying gaps, strengths, and opportunities for enhancing participation assessment in stroke rehabilitation.

**Method:** A comprehensive search of keywords related to stroke, participation, and assessment was conducted across multiple databases, including Scopus, Embase, Medline, Cochrane Library, PEDro, and OTseeker. The review process was conducted based on the PRISMA–Consensus-based Standards for the Selection of Health Measurement Instruments (COSMIN) guidelines. The COSMIN checklist was utilized to investigate the psychometric properties of the studies, and the results were synthesized narratively.

**Results:** Thirty-seven studies were ultimately included in the review after the full-text evaluation. The review identified 10 PROMs, including the Functional Behavior Profile, Meaningful Activities Participation Assessment, Nottingham Extensive Activities of Daily Living Scale, Rivermead Mobility Index, Reintegration to Normal Living Index, The Subjective Index for Physical and Social Outcome, and Stroke Impact Scales. While these tools are developed with appropriate psychometric properties and focus on varying dimensions of survivors' participation, significant gaps remain in fully addressing their comprehensive needs. Specifically, the tools often overlook contextual differences and fail to adequately assess satisfaction in daily activities.

**Conclusion:** The identified gaps illustrate the need for more comprehensive measures that accurately capture stroke survivors' diverse experiences. Improving these assessments is essential for informing clinical practice and rehabilitation strategies, ultimately enhancing patient engagement, satisfaction, and quality of life and improving rehabilitation outcomes for stroke survivors.

## 1. Introduction

The publication of the International Classification of Functioning, Disability and Health (ICF) in 2001 represented a paradigm shift in the conceptualization of health, moving away from the limitations of its predecessor, the International Classification of Impairment, Disability, and Handicap (ICIDH) established in 1980 [1, 2].

This innovative framework encompasses a comprehensive view, integrating nearly every aspect of an individual's life, including functional and contextual factors, into the definition of health. This approach significantly diverges from the traditional medical model, promoting a dynamic perspective on health that distinguishes an individual's health status from their medical diagnosis [3]. In this dynamic approach, “participation” plays an important role. According to the ICF, it means involvement in life situations [4].

Due to the importance of participation in people's health and quality of life, increasing people's participation in activities and society has become the primary goal of rehabilitation programs [5]. In the field of occupational therapy, which is a crucial discipline dedicated to the rehabilitation of stroke survivors, the concepts of occupational performance and participation closely correspond to the definitions outlined by the ICF. Occupational performance, as articulated in the *Occupational Therapy Practice Framework: Domain and Process* (OTPF-4), encapsulates an individual's capacity to engage in and accomplish meaningful activities and tasks within the context of daily life [6].

Subsequently, numerous models and assessment tools have been developed to evaluate both occupational performance and client participation. Among these, the Canadian Model of Occupational Performance and Engagement (CMOP-E) stands out as a leading framework, emphasizing the dynamic relationship between the individual (person), their activities (occupation), and their environment. Such an integrative perspective acknowledges the various restrictions that may impact participation and functional capacity within an individual's life [7].

Restrictions in participation, functional autonomy, and occupational performance in everyday activities occur across various conditions and disabilities [8]; one of these conditions is stroke.

Stroke is recognized as one of the leading causes of disability. In the 11th edition of the *International Classification of Diseases* (*ICD-11*), stroke falls under cerebrovascular diseases, encompassing a range of neurological disorders related to the brain's blood vessels. This category includes intracerebral hemorrhage, subarachnoid hemorrhage, ischemic stroke, and stroke of unknown origin [9].

In 2021, there were an estimated 10.3 million new reported cases of stroke, 160.5 million individuals living with stroke-related disabilities, and 7.3 million stroke-related deaths; this makes stroke the third leading cause of death and the fourth leading cause of disability worldwide [10]. The high prevalence of disability due to stroke results in significant limitations in participation and functional independence in daily life activities for those affected [11].

Valid and reliable assessment tools are necessary to appropriately set goals and plan interventions targeting participation [4].

Assessing participation, alongside its diverse dimensions, is crucial for designing rehabilitation programs for stroke survivors [3, 11]. However, it is important to note that no single tool comprehensively addresses all domains of participation [12].

Eyssen et al. [13] conducted a systematic review evaluating 103 tools designed to assess participation across various dimensions, including barriers, levels of engagement, and satisfaction in areas such as work, social life, home, leisure, transportation, and shopping. However, most of these tools focused on limited aspects of participation, often neglecting family life and financial activities. Furthermore, they primarily aimed to identify problems and measure participation “success” rather than gauging “satisfaction.” Similarly, the systematic review by Tse et al. [12] identified 36 tools specifically for stroke patients, with five being the most commonly used. Despite their contributions, none of these tools provided a comprehensive evaluation of all dimensions of participation, and given that more than a decade has passed since this study, newer tools may have been developed that were not assessed in their review. In addition, Hotta et al. [14] conducted a scoping review that examined 20 measurement tools for assessing participation in individuals with cancer. Their findings revealed that most tools were not specifically designed to evaluate dimensions of participation, and only four considered aspects such as value, satisfaction, and meaning. Notably, none of the tools was specifically developed for individuals with cancer.

Despite the inherent limitations associated with participation assessments, it is crucial to acknowledge that nearly all of these measures exhibit notable strengths [12–14]. This underscores the necessity for a comprehensive review that can assist professionals in selecting among these measures, considering their respective advantages and disadvantages.

Considering the participation dimensions (i.e., satisfaction, frequency, and meaningfulness), which are inherently client-centered and emphasize each individual's unique perspective and cultural values, it is necessary that the chosen assessment tool or outcome measure be based on patient self-report. This approach ensures that evaluations are grounded in the clients' subjective experiences, offering a more nuanced and comprehensive understanding of their engagement and outcomes [15].

The subjective experiences and emotions of stroke survivors regarding their participation in daily activities and social roles are far more intricate than what can be captured through objective clinical assessments. Many survivors express feelings of frustration and loss as they face the challenges of re-engaging in activities they once enjoyed, which may now seem daunting or even unattainable. They often report a heightened awareness of their health responsibilities, fostering a strong desire to prevent future strokes while simultaneously grappling with the emotional and psychological repercussions of their condition. Furthermore, qualitative findings indicate that survivors perceive their participation not merely as a return to physical abilities, but as a multidimensional experience that encompasses emotional well-being, social connections, and a sense of identity [16].

The last review of participation assessments among stroke survivors was carried out over a decade ago and focused solely on the five most frequently used tools [12]. As a result, the challenge of choosing the most appropriate tool that meets their specific clinical needs and therapeutic goals for current clinicians remains. The importance of assessing participation in this population further underscores this challenge.

Therefore, this systematic review is aimed at evaluating the patient-reported outcome measures (PROMs) designed for individuals with stroke, focusing on measuring participation and its dimensions.

## 2. Methods

This systematic review followed the PRISMA-COSMIN guideline for reporting outcome measurement instrument systematic reviews [17].

### 2.1. Search Strategy

The keywords related to stroke (Stroke, Cerebrovascular, Ischemi⁣^∗^, haemorrhag⁣^∗^, Intracerebral haemorrhage, and Subarachnoid haemorrhage) which were chosen based on *ICD-11* subcategories, participation (Participation, Engagement, Involvement, Performance, Occupational performance, Occupation, and Function) and assessments (Assessment, Scale, Inventory, List, Questionnaire, Index, Tool, Checklist, Instrument, and Outcome measure), were searched in Scopus, Embase, Medline, Cochrane library, PEDro, and OTseeker databases using Boolean operators “OR” and “AND” in May and June 2024 (Table 1).

### 2.2. Eligibility Criteria

Studies were included if they were published in peer-reviewed English journals; involved the development, translation, or assessment of PROMs' psychometric properties; targeted individuals with a documented history of stroke; and explored dimensions of participation as conceptualized within the ICF framework.

Case reports, reviews, qualitative or theoretical research, observation-based or interview-based measures, and studies that examined different types of neurological diseases other than stroke, the strengths and weaknesses of the measures, social participation exclusively, and studies for which full texts were not accessible were excluded.

### 2.3. Review Process

**Figure 1:**
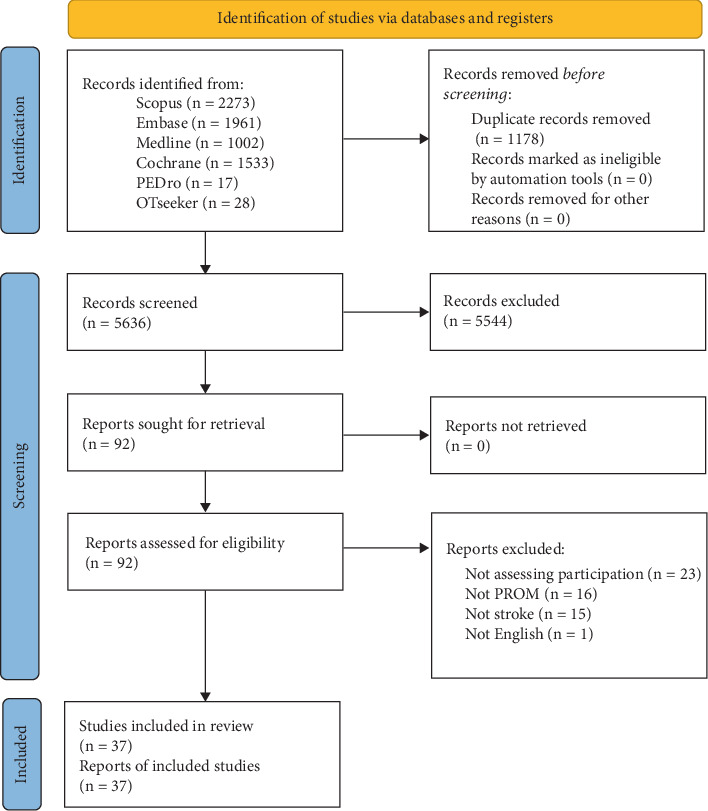
PRISMA 2020 flow diagram for new systematic reviews which included searches of databases and registers only.

The initial results, including 6838 studies, were first entered into Mendeley software (https://www.mendeley.com/), and duplicate studies were removed; the remaining studies underwent further examination using the Rayyan website for systematic reviews (https://rayyan.ai). After removing duplicates, each study was independently screened by the first and second reviewers based on its title and abstracts. In the next step, the full texts of 92 studies were assessed for eligibility, and 10 PROMs and their translations (37 studies) were included, as detailed in Figure 1. Any disagreements at any stage of the review process were resolved through discussion, and if agreement was not reached, the final decision was made by a third independent reviewer.

### 2.4. Data Extraction

According to the COSMIN risk of bias checklist [18], this study categorized the measurement psychometric properties into two domains: reliability (encompassing test–retest, internal consistency, interrater, and intrarater reliability) and validity (including face, content, construct, and criterion validity). Both discriminant (divergent) and convergent validity were considered within the construct validity, whereas concurrent validity was regarded as criterion validity [19].

In addition to psychometric properties, the characteristics of the studies included in this review (i.e., the target population, mode of administration, recall period, number of items, subscales, score ranges, scoring methodology, original language, and available translations) were systematically extracted. This extraction was conducted exclusively for the original studies that presented the initial versions of the included measures.

The findings of this review were synthesized based on Popay et al.'s [20] narrative synthesis method.

### 2.5. Risk of Bias

The COSMIN risk of bias is used to assess the methodological quality of a study or to compare the attributes of different measurement tools within a systematic review [18].

## 3. Results

Following the review process (Figure 1), 10 PROMs were identified that evaluate the participation of poststroke patients in their recovery or discharge planning. The original versions of these PROMs, along with their translations and psychometric properties for poststroke patients, were included, bringing the total number of studies included to 37—comprising 10 original versions and 27 eligible translated versions.

The characteristics of the PROMs are detailed in Table 2.

### 3.1. Risk of Bias and Psychometric Properties of PROMs

The findings concerning the psychometric properties of the PROMs are outlined in Table 3.

### 3.2. PROMs

#### 3.2.1. The Functional Behavior Profile (FBP)

The FBP, developed by Baum et al. [21], is a clinical tool designed to help determine placement or discharge decisions for poststroke patients. This assessment tool has been validated and proven reliable for use with English-speaking patients who have suffered a stroke. The FBP comprises 27 items, each evaluating the individual's ability to perform tasks, engage in social interactions, and solve problems. Items are scored on a scale from 0 to 4, with assessments conducted either through self-reporting by the patient and caregiver or via an interview [21].

#### 3.2.2. Meaningful Activities Participation Assessment (MAPA)

The MAPA represents a rigorously designed checklist survey developed by Eakman et al. [22] within the United States to measure the frequency and personal meaningfulness attributed to 28 varied activities by older adults. This measure enables participants to articulate the extent of their engagement in these activities alongside the depth of meaning they derive from such involvement. The assessment operates on a scoring schema ranging from 0 to 4 for each item, where higher scores indicate a greater level of perceived meaningful activity participation, as self-reported by the respondents. Since its development, the MAPA has been subject to linguistic adaptation and translated into Chinese and Persian, and its psychometric properties have been assessed in Taiwan and Iran [22, 31, 32].

#### 3.2.3. The Nottingham Extensive Activities of Daily Living (NEADL) Scale

Developed by Nouri and Lincoln [23] in the United Kingdom, the NEADL scale serves as an evaluative measure for assessing the capacity of stroke patients to independently undertake activities of daily living (ADLs) within a home setting. This scale has 22 items, grouped into five domains: mobility, kitchen activities, domestic activities, leisure activities, and financial management, to determine levels of patient independence [23]. The NEADL has been translated into several languages, including Chinese, Turkish, and Welsh, and its psychometric properties have been assessed in studies conducted in Taiwan [33] and Türkiye [34], affirming its applicability in diverse cultural contexts.

#### 3.2.4. Rivermead Mobility Index (RMI)

The RMI is a quick tool developed by Collen et al. [24] and designed to assess mobility in neurologically impaired patients across various clinical settings, especially after stroke. It consists of 15 questions and primarily measures a patient's ability to move independently [24]. The RMI is valid and reliable in clinical practice in different contexts, including Brazil [35], Denmark [36], Italy [37], Germany [38], the Netherlands [39], and the United Kingdom [24], making it helpful in evaluating mobility disability and guiding treatment interventions.

#### 3.2.5. Reintegration to Normal Living Index (RNLI)

The RNLI is a comprehensive measure for assessing the extent of global functional reintegration in individuals after a stroke, following a debilitating illness, or experiencing severe trauma. Initially developed by Wood-Dauphinee et al. [25] within the Canadian context, the measure was designed to accommodate French- and English-speaking populations. The RNLI contains 11 items, categorically distributed across five domains: mobility, self-care, daily activities, recreational activities, and family roles. Each item on the RNLI is rated on a scale from 1 to 10, with higher scores indicating more successful reintegration into prior functional roles and community life [25].

Furthermore, the measure has been subject to linguistic adaptation, with translations available in Chinese and Spanish. Psychometric evaluations conducted in China and Spain proved this PROM valid and reliable [40, 41].

#### 3.2.6. The Subjective Index for Physical and Social Outcome (SIPSO)

The SIPSO is a 10-item self-report questionnaire developed by Trigg and Wood [26] in the United Kingdom and designed to assess an individual's ability to reintegrate into a normal lifestyle. It focuses on physical and social functioning and measures the quantity and quality of activities and interactions, reflecting personal satisfaction with functioning [26].

The SIPSO assesses two main domains: physical functioning/mobility and social/emotional functioning. It includes activities related to hobbies, social interactions, and responsibilities both in-home and outside, as well as environmental factors like physical and financial dimensions. These domains reflect the individual's subjective perceptions of physical and social integration into daily life. This measure has been translated into Chinese (Cantonese) and Portuguese. In addition to the United Kingdom, its psychometric properties have been evaluated in Hong Kong (China) for poststroke patients [42, 43].

#### 3.2.7. Stroke Impact Scale (SIS)

The SIS was initially developed by Duncan et al. [27] as SIS 2.0 to provide a comprehensive measure of stroke outcomes, encompassing 64 items across eight domains. These domains include strength, hand function, ADL/IADL, mobility, communication, emotion, memory and thinking, and participation. The SIS 2.0 utilizes a scoring algorithm where lower scores indicate a more significant impact on functioning. Respondents answer questions based on their experiences; the scores reflect the degree of impairment, disability, or handicap resulting from a stroke. The scores obtained can be used to evaluate health and quality of life changes over time [27]. Furthermore, SIS 2.0 has been translated into French, German, and Portuguese. Its psychometric properties have been examined in various countries, including the United States [27], Australia [44], France [45], Germany [46], Brazil [47], and Portugal [48], demonstrating its validity and reliability in different languages and cultural contexts. In 2003, SIS 2.0 was updated, evolving into SIS 3.0 [28]. Subsequently, its psychometric properties were evaluated in a variety of global settings, including Morocco [49], Italy [50], India [51], Nigeria [52], South Korea [53], Uganda [54], Brazil [55], Türkiye [56], and Pakistan [57], consistently affirming its validity and reliability. In Malaysia, it was also found to possess strong content and linguistic validity [58]. Additionally, shorter versions of SIS, such as SIS-16 [29] and SIS-SF [30], have been developed. SIS-16 has been proven to be valid and reliable in Australia [44], Pakistan [57], and the United States [29]. At the same time, SIS-SF is valid and reliable for use in the United Kingdom [30] and Germany [59] for English and German speakers.

## 4. Discussion

### 4.1. Summary and Interpretation of Findings

This systematic review identified 10 PROMs, including FBP, MAPA, NEADL, RMI, RNLI, SIPSO, SIS 2.0, SIS 3.0, SIS-16, and SIS-SF. The PROMs were developed to assess poststroke patients' activity participation, and the translations' psychometric properties were also assessed for this target population. Excluding the FBP, all PROMs demonstrated strong validity and reliability across various global contexts. The PROMs exhibited comparable internal consistency, test–retest reliability, and construct and criterion validity across different countries and languages, underscoring their robustness in diverse settings.

A PROM is highly recommended, given the significance of adopting a client-centered framework within rehabilitation contexts. The PROMs enable clinicians to consider and integrate the patient's preferences, satisfaction levels, and perceptions of their functional status, thereby enhancing the overall effectiveness of the treatment and recovery [60]. To be used as an assessment by a clinician, a PROM should be easy for the patient to understand, have an appropriate number of items, have good psychometric properties, and have the possibility to assess more dimensions of participation [61].

Among the included PROMs, SIS 3.0 is the most internationally known measure, with nine available translations in addition to the original English version [58]. It also includes English (United Kingdom) and English (United States) versions. Although SIS 3.0 is not the oldest PROM, it covers many different domains related to the daily living of the patients. It provides valuable information about most aspects of the patient's life that can be affected by a stroke. The disadvantage of this measure is the poor psychometric properties of the “emotion” domain in most contexts and the long administration time compared to other PROMs.

MAPA is the only PROM specifically designed to evaluate the participation of individuals after stroke. It offers valuable insights into various aspects of the patient's life. However, it does not categorize this information into different activity-based subscales, which may make it challenging for assessors to identify the specific areas of activity affected. Its primary objectives are to assess the frequency and meaningfulness of activities to the individual. The limited availability of translated versions worldwide may be attributed to the fact that MAPA is the most recently published PROM among the measures included.

While all PROMs can be used to guide clinicians on the intervention process, two measures, RNLI and SIPSO, were developed purposefully to evaluate the patient's reintegration into normal life and their satisfaction with functioning. Although these two measures have the same number of items and the same psychometric properties in most contexts, SIPSO shows more diverse subscales that can provide more information about different aspects of the patient's physical and social activities.

FBP was originally designed to evaluate a patient's readiness for discharge; however, the limited availability of translations and the insufficient evidence supporting its psychometric properties render it less suitable for use among practitioners. Its items mainly focus on performing ADL tasks similar to NEADL. Comparing FBP and NEADL, NEADL has more available translations and better psychometric properties; it also needs less time to complete. NEADL also provides information in a more organized way, covering five integral domains of ADLs.

While all PROMs address mobility-related activities, the RMI specifically evaluates the functional mobility of individuals following the onset of disability. It has been translated into six other languages, enhancing its validity and reliability across diverse contexts.

Considering the critical role of participation in health, as delineated by the ICF, the inclusion of PROMs is pivotal for clinicians and healthcare professionals to ascertain an individual's health status through their own perceptions or to identify the necessity for intervention. Among the included PROMs, the SIS 3.0 emerges as the most linguistically accessible measure, boasting the highest number of translations. It explicitly addresses activities impaired by stroke, ADLs, and social participation. Notably, it accommodates individuals unable to independently complete the measure, offering the option for completion via interview. Furthermore, SIS 3.0 demonstrates robust psychometric qualities across most of its subscales. Given these attributes, SIS 3.0 is recommended as an optimal PROM in scenarios where the evaluator and patient can allocate sufficient time, particularly when the patient's life is extensively impacted.

It is essential to consider the role of leisure in a patient's daily life, as it can help reduce the risk of depression, a common psychological effect of stroke [62, 63]. Currently, the only PROMs that assess leisure are MAPA and NEADL. If the primary aim is to evaluate a patient's independence in daily activities, including leisure, and if independence in each area of activity is crucial, NEADL is the recommended choice. On the other hand, if the evaluation goals involve assessing activity participation, meaningfulness, or frequency of activities without considering an area of activities, then MAPA is the recommended PROM.

Although, in most cases, the stroke impacts both physical and social activities [64], RMI only assesses mobility-related activities in a short time; considering its availability across different contexts worldwide, it is a suitable PROM when time is a matter and when only the functional mobility of the individual needs to be assessed.

### 4.2. Strengths and Limitations

In this systematic review, particular emphasis was placed on the client-centered approach and the ICF model of health. Although the eligibility criteria were carefully set to encompass all pertinent studies and self-report measures developed for stroke survivors, there is always a risk of selection bias in systematic reviews. The criteria for selecting studies might have inadvertently excluded relevant PROMs or studies, potentially impacting the comprehensiveness of the findings. For example, five translated versions of the included measurement instruments were excluded due to predefined eligibility criteria. These include the Italian FBP, which its psychometric properties have not been evaluated for stroke survivors; the Portuguese SIPSO, which was presented as a conference paper and lacks peer review; the Danish RMI, which has not been assessed for stroke survivors; and the SIS 3.0 and the Spanish SIS-16, which their results were not published in English.

Adhering to the PRISMA-COSMIN guideline, data were extracted based on the COSMIN risk of bias checklist, which comprehensively addresses the measures' characteristics and psychometric properties. This approach provided valuable insights into the reliability and validity of these tools, significantly contributing to poststroke care and rehabilitation.

It is crucial to consider the impact of stroke on ADLs and social participation. However, assessing participation in leisure activities and work performance is also important. Among the measures included, only SIPSO assesses all of these aspects. Furthermore, while almost all included measures evaluate the frequency and satisfaction of activity participation, only MAPA fully addresses the core components of participation, including value, meaningfulness, and satisfaction.

Across diverse cultural contexts, varying values and beliefs can imbue different meanings to participation and satisfaction with activity, especially in the case of self-report assessment tools [6]. While many PROMs have demonstrated validity and reliability across different cultural settings (e.g., China, Türkiye, Pakistan, the United States, and the United Kingdom), the influence of distinct cultural values remains a concern. As such, the need to develop culturally sensitive tools tailored to each specific context is underscored. This ensures the accurate capture of the intricacies of participation and satisfaction among stroke survivors.

### 4.3. Recommendations and Implications for Future Studies

In light of the ICF's conceptualization of participation, all included PROMs investigate stroke survivors' participation in ADLs and other affected activities by stroke. SIS 3.0, SIS-SF, and SIS-16, alongside MAPA, were introduced after the 2001 ICF publication. Among these, MAPA is the only measure aimed at measuring participation, underscoring its significance in patient health and quality of life. Thus, it is recommended that more targeted measures be developed to assess participation and all its dimensions in poststroke patients.

Considering global cultural diversity, it is imperative for communities with different activity participation values and perspectives to develop new measures that align with their cultural contexts and values.

It is further recommended that future measures extend their focus to encompass a broader array of activities impacted by stroke, notably in the domains of leisure and work performance. These dimensions were not addressed in the measures examined within this review.

Future research should prioritize a thorough evaluation of the psychometric properties of interview-based PROMs, ensuring their reliability and validity across a wider spectrum of neurological disorders. This would contribute to improved diagnostic accuracy and ultimately enhance patient outcomes.

## 5. Conclusions

This review highlights 10 PROMs for stroke survivors, focusing on a client-centered approach and the ICF model. SIS 3.0 is notable for its accessibility, comprehensive coverage, and strong psychometrics, making it a top choice for detailed assessments. However, PROM selection should be tailored to specific evaluation goals, considering factors like focus on daily living, leisure, or functional mobility, and practical issues like administration time and language availability. NEADL is best for assessing independence in daily activities, while MAPA offers insights on poststroke participation, albeit with a limited linguistic range. RMI is recommended for quick functional mobility assessments.

## Figures and Tables

**Table 1:** Search strategy.

Search terms (AND, OR)	Stroke OR Cerebrovascular OR Ischemi⁣^∗^ OR haemorrhag⁣^∗^ OR Intracerebral haemorrhage OR Subarachnoid haemorrhage AND Participation OR Engagement OR Involvement OR Performance OR Occupational performance OR Occupation OR Function AND Assessment OR Scale OR Inventory OR List OR Questionnaire OR Index OR Tool OR Checklist OR Instrument OR Outcome measure
The type of studies searched	Journal articles
Searched fields	All fields (abstract, title, journal name, author name, author affiliations, etc.)
Date limit	None
Language limit	English results only
Types of included studies	Only methodological studies

**Table 2:** Characteristics of the included PROMs.

**PROMs (reference to the first article)**	**Target population**	**Mode of administration**	**Recall period**	**Number of items/subscales**	**Range of scores/scoring**	**Original language**	**Available translations**
1. FBP [21]	Stroke, Alzheimer's	Self-report Interview based	10–20 min	27/none	0–4/0–108	English	Italian^a^
2. MAPA [22]	Stroke	Self-report	Not defined	28/ - Frequency of participation - Meaningfulness of participation	0–6 for repetitions 0–4 for meaningfulness/0–672	English	Chinese, Persian
3. NEADL [23]	Stroke	Self-report Postal report	10 min	22/ - Mobility - Kitchen activities - Domestic activities - Leisure activities - Financial management	0–3/0–66	English	Chinese, Turkish, Welsh
4. RMI [24]	Stroke, head injury, spinal cord injury, and multiple sclerosis	Self-report Observation	3 to 5 min	15/none	0 and 1/0–15	English	Italian, Danish^a^, Dutch, German, Portuguese
5. RNLI [25]	Stroke	Self-report Postal report Interview based Proxy report	< 10 min	11/ - Mobility - Self-care - Daily activity - Recreational activity - Family roles	0–10/0–110	English, French	Chinese, Spanish
6. SIPSO [26]	Stroke	Self-report	Not defined	10/ - Physical functioning - Social/emotional functioning	0–4/0–40	English	Turkish, Chinese (Cantonese), Portuguese^a^
7. SIS 2.0 [27]	Stroke	Self-report Interview based Proxy report	15–20 min	64/ - Strength - Hand function - ADL and IADL - Mobility - Communication - Emotion - Memory and thinking - Participation	1–5/0–100	English	French, German, Portuguese
8. SIS 3.0 [28]	Stroke	Self-report Interview based Proxy report	15–20 min	59/ - Strength - Hand function - ADL/IADL - Mobility - Communication - Emotion - Memory and thinking - Participation and role function	1–5/0–100	English	Arabic (Darija), Italian, Gujarati, Korean, Luganda, Malay, Portuguese, Turkish^a^, Urdu
9. SIS-16 [29]	Stroke	Self-report Interview based Proxy report	5–10 min	16/ - ADL/IADL - Mobility - Hand function	1–5/0–100	English	Spanish^a^
10. SIS-SF [30]	Stroke	Self-report Interview based Proxy report	Not defined	8/ - Strength - Hand function - ADL/IADL - Mobility - Communication - Emotion - Memory and thinking - Participation and role function	1–5/0–100	English	German

Abbreviations: FBP, The Functional Behavior Profile; MAPA, Meaningful Activities Participation Assessment; NEADL, The Nottingham Extensive Activities of Daily Living; RMI, Rivermead Mobility Index; RNLI, Reintegration to Normal Living Index; SF, Stroke Impact Scale; Short, Form; SI, S; SIPSO, The Subjective Index for Physical and Social Outcome; SIS, Stroke Impact Scale.

^a^Excluded.

**Table 3:** Psychometric properties of the included PROMs.

**PROMs**	**Language/country**	**Validity**					**Reliability**			
		**Study population/** **n**	**Face**	**Content**	**Construct**	**Criterion**	**Test–retest [ICC]**	**Internal consistency [Cronbach's alpha]**	**Interrater [kappa/ICC]**	**Intrarater [ICC]**
FBP	English/United States	Stroke/45	0	0	?	?	0	[0.86]	0	0

MAPA	Chinese/Taiwan	Stroke/146	0	0	0	The correlations with the established tools (EMAS, NPS, Interest Checklist, ACS, CES-D, SF-36) were significant	[0.94]	?	0	0
	English/United States	Stroke/154	+	+	0	The correlations with the established tools (LSI-Z, SWLS, PIL, EMAS, CES-D, SF-36) were significant	?	[0.77 to 0.89]	0	0
	Persian/Iran	Stroke/107	0	0	*Convergent validity:* The correlations with the established tools (PIL-SF, CES-D, LSI-Z, SWLS, SF-36) were significant	0	[0.92]	[0.79]	0	0

NEADL	Chinese/Taiwan	Stroke/153	+	0	The correlations with BI were significant [+]	0	0	?	0	0
	English/United Kingdom	Stroke/52	+	0	0	*Concurrent validity:* The correlations with the established tools (LHS, SF-36) were significant [+]	0	[0.9]	0	0
	Turkish/Türkiye	Stroke/60	0	0	The correlations with BI were significant	0	[0.84 to 0.97]	[0.74 to 0.97]	0	0

RMI	English/United Kingdom	Stroke/39			The correlation with BI was significant	*Concurrent validity:* The correlations with the established tools (Modified RMI, STREAM) were significant	[0.96]	[0.96]	[0.92]	
	Italian/Italy	Stroke/308	0	0	Rasch analysis supports the validity [+]	0	0	?	0	0
	Dutch/Netherlands	Stroke/420	0	0	Significant correlation with the Dutch BI [+]	0	0	[0.97]	0	0
	Portuguese/Brazil	Stroke/95	0	+	0	*Concurrent validity:* The correlations with the established tools (BI, NEADL) were significant	0	[0.96 to 0.99]	0	0
	German/Germany	Stroke/197	0	0	0	*Concurrent validity:* The correlations with the established tools (10-MWT, Motor FIM) were significant [+]	0	0	[?]/significant correlations correlation of *r* = 0.98 and a total agreement of 75% [+]	0

RNLI	Chinese/China	Stroke/75	0	0	*Convergent validity:* The correlations with the established tools (FAI, PWI) were significant [+]	0	[0.87]	[0.92]	[0.41 to 0.60]	0
	English and French/Canada	Stroke/not defined	0	0	*Convergent validity:* The correlations with the established tools (FAI, SF-36, BI, HADS) were significant [+]	0	[0.38 to 0.92]	[0.84]	[ICC = 0.36–0.55]	0
	Spanish/Spain	Stroke/35 and total knee arthroplasty/35	+	+	*Convergent validity:* The correlations with the established tools (6-MWT, SF-36) were significant [+] *Discriminant validity:* The index effectively distinguishes between different domains as intended [+]	0	[0.55]	[0.86 to 0.94]	0	0

SIPSO	Cantonese/China	Stroke/92	+	+	*Convergent validity:* The correlations with the established tools (FAI, PWI) were significant [+]	0	[0.87]	[0.92]	[Kappa = 0.41–60] for most items	0
	English/United Kingdom	Stroke/260	0	0	The correlations with the established tools (FAI, NHP, WDI, BI) were significant [+]	0	[0.4 to 0.7]	[0.92]	0	0

SIS 2.0	English/Australia	Stroke/74	0	0	*Convergent validity:* Strong correlation with established measures (SDS and WHOQOL-BREF) in all domains except emotion and ADL/IADL [+] *Discriminant validity:* Adequate item validity [+]	0	?	[> 0.90] except the emotion domain ( = 0.80)	0	0
	English/United States	Stroke/91	0	0	*Convergent validity*: Modest to strong correlations with established measures (BI, FIM, FM, MMSE, NIH Stroke Scale, SF-36, Duke Mobility Scale, and GDS), with coefficients ranging from 0.44 to 0.84 [+] *Discriminant validity:* Six domains displayed significant differences across groups defined by the Rankin scores [+]	0	[0.7 to 0.92] except for emotion (0.57)	[0.83 to 0.90]	0	0
	French/France	Stroke/288	0	0	The grouping of items in the eight domains (except for one ADL and three emotion items) appeared adequate [+] *Convergent validity:* High correlations between all domains and the established measures (BI, HADS, Duke Health Profile) with a coefficient exceeding 0.54 [+]	0	[0.65 to 0.95]	[0.89]	0	0
	German/Germany	Stroke/196	0	0	No significant DIF was found for any items [+]	0	0	[0.67 to 0.92]	0	0
	Portuguese/Brazil	Stroke/40	0	0	0	0	0	[0.75 to 0.99]	[ICC = 0.73 to 0.99, *p* < 0.0001]	[0.75 to 0.99, *p* < 0.0001]
	Portuguese/Portugal	Stroke/498	0	0	Subjects without complications during hospitalization scored significantly higher on most SIS 2.0 domains [+]	Adequate correlations between the domains and related measures (CMSA and health status questionnaires) [+]	[0.70 to 0.95]	[0.83 to 0.96]	0	0

SIS 3.0	Arabic (Darija)/Morocco	Stroke/102	0	+	*Convergent validity:* A strong correlation between items and their dimensions, with coefficients exceeding 0.4 [+] *Divergent validity:* The results showed a significant difference according to the type of stroke in the scores of different domains [?]	0	[0.778 to 0.921]	[0.70 to 0.945]	0	0
	English/United Kingdom	Stroke/19	0	0	*Convergent validity:* All domains were highly correlated with EQ-5D (*ρ* = 0.77 to 0.94, *p* < 0.001) [+] Factor analysis supports the construct validity [+]	0	0	[0.86 to 0.95]	0	0
	English/United States	Stroke/696	0	0	Factor analysis supports the construct validity [+]	0	0	[0.70 to 0.90]	0	0
	Gujarati/India	Stroke/26	+	+	0	*Concurrent validity:* Moderate to weak correlations with established scales (NIHSS, MMSE, FIM, and STREAM) [+]	0	0	0	0
	Hausa/Nigeria	Stroke/140	+	+	*The discriminant validity:* Sufficient validity between the constructs [+]	0	0	[0.74 to 0.92] except for emotion ( = 0.58)	0	0
	Italian/Italy	Stroke/392	0	+	0	*Concurrent validity:* Correlations with the scales (SF-36, BI, MMSE, HADS, NIHSS, and IADL) were almost all significant [+]	[0.79 to 0.93]	[0.89 to 0.98]	0	0
	Korean/South Korea	Stroke/70	0	0	0	*Concurrent validity:* All domains except emotion were highly correlated with the scales (SF-36, MMSE, MBI, NIHSS) [+]	[?] Spearman's rho was above 0.5, except for social participation and mobility	[0.719 to 0.957]	0	0
	Luganda/Uganda	Stroke/25	0	+	No items were demonstrating DIF except for ADL and participation domains [+]	0	0	[0.90 to 0.96] except for emotion ( = 0.75)	0	0
	Malay/Malaysia	0	+	+	0	0	0	0	0	0
	Portuguese/Brazil	Stroke/174	0	0	*Convergent validity:* All domains were significantly correlated with related scales (NIHSS, MMSE, BI, LIADLS, modified Rankin Scale, GDS, HADS) [+] *Discriminant validity:* All domains except for “memory” effectively distinguished between most health conditions [+]	0	[0.48 to 0.94]	[0.94] except for emotion ( = 0.49)	0	0
	Urdu/Pakistan	Stroke/90	0	0	*Convergent validity:* The correlations with the established tools (SF-36, BI, MRS, STREAM, HADS, MMSE) were significant [+]	0	[0.77 to 0.95]	[0.83 to 0.93] except for emotion ( = 0.68)	0	0

SIS-16	English/Australia	Stroke/74	0	0	*Convergent validity:* The correlation with related tools (SDS and the WHOQOL-BREF) was significant [+] *Divergent validity:* Items within the same domain have higher correlations with their own domain than others [+]	0	0	[0.92]	0	0
	English/United States	Stroke/621	0	+	Factor analysis supports the construct validity [+]	*Concurrent validity:* Correlations with the scales (BI, BRS) were significant [+]	[> 0.80]	0	0	0
	Urdu/Pakistan	Stroke/90	0	0	*Convergent validity:* The correlations with the established tools were significant [+]	0	[0.93]	[0.85]	0	0

SIS-SF	English/United Kingdom	Stroke/151	0	0	*Convergent validity:* The correlations with the established tools (EQ-5D, SIS index) were significant [+]	0	0	[0.89]	0	0
	German/Germany	Stroke/150	0	0	The correlations with the established tools (SIS 2.0, EQ-5D, NIHSS, MMI) were significant [+]	0	[0.88]	[0.89]	0	0

Abbreviations: 6-MWT, 6-min walk test; 10-MWT, 10-min walk test; ACS, Activity Card Sort; BI, Barthel Index; CES-D, Center for Epidemiologic Studies Depression Scale; CMSA, Chedoke-McMaster Stroke Assessment; DIF, Differential Item Functioning; EMAS, Engagement in Meaningful Activities Survey; EQ, EuroQol; FAI, Frenchay Activities Index; FIM, Functional Independence Measure; FM, Fugl-Meyer; GDS, Geriatric Depression Scale; HADS, Hospital Anxiety and Depression Scale; LIADLS, Lawton Instrumental Activities of Daily Living Scale; LSI-Z, Life Satisfaction Index-Z; MBI, Modified Barthel Index; MMI, Morton Mobility Index; MMSE, Mini-Mental State Examination; NHP, Nottingham Health Profile; NIHSS, National Institutes of Health Stroke Scale; NPS, Norling, Pettersson, Selander; PIL, Purpose-in-Life Test; PWI, Personal Wellbeing Index; STREAM, Stroke Rehabilitation Assessment of Movement; SWLS, Satisfaction with Life Scale; WHOQOL, World Health Organization Quality of Life; WPI, Wakefield Depression Inventory; ZDS, Zung Self-Rating Depression Scale.

## Data Availability

Data sharing is not applicable to this article as no datasets were generated or analyzed during the current study.
